# Characterizing short‐term linear and nonlinear heart rate variability in typically developing children and adolescents

**DOI:** 10.14814/phy2.71126

**Published:** 2026-10-04

**Authors:** Alexs A. Matias, Michele N. D'Agata, Krista M. Szymanski, Evan C. Ciecko, Melissa A. Witman

**Affiliations:** ^1^ Department of Kinesiology and Applied Physiology University of Delaware Newark Delaware USA

**Keywords:** complexity, development, kids, neurocardiac function, pediatric

## Abstract

Linear heart rate variability (HRV) quantifies heart rate (HR) changes due to stochastic processes while nonlinear HRV captures the complexity of the HR structure. Although linear HRV in childhood and adolescence has been explored, little is known about nonlinear HRV. The present study characterized short‐term linear and nonlinear HRV in typically‐developing children (7–12 years, *n* = 35) and adolescents (13–17 years, *n* = 29). HRV during quiet rest was assessed using a three‐lead electrocardiogram for 5‐min and analyzed using linear (time and frequency domain) and nonlinear (Poincáre analysis, approximate [ApEn] and sample entropy [Sampen], and detrended fluctuation analysis [DFA]) metrics. A greater mean RR time‐interval was observed in adolescents (*p* = 0.007). Time‐domain HRV was not different between children and adolescents, although sex differences were observed, such that females had lower linear HRV (*p* < 0.05, $\hat{\theta }$ > 62); namely, standard deviation of the normal sinus interbeat intervals (SDNN) and root mean square of the successive differences between the normal interbeat intervals (RMSSD). ApEn was lower in adolescent males (*p* < 0.001, $\hat{\theta }$ = 86). Short‐term scaling DFA was also lower in adolescents (*p* = 0.023, $\hat{\theta }$ = 64). Poincaré analysis revealed higher nonlinear short‐term (*p* < 0.003, $\hat{\theta }$ = 68) and long‐term variability (*p* < 0.001, $\hat{\theta }$ = 83) in males. Nonlinear metrics correlated with linear indices of parasympathetic tone. Collectively, age and sex differences were observed in nonlinear HRV in children and adolescents. Future research is needed to elucidate the mechanisms underlying nonlinear HRV during development and how these processes are altered in childhood disorders.

## Graphical abstract

Nonlinear heart rate variability differed by age and sex in typically developing youth. Nonlinear measures were associated with parasympathetic indices. Figure generated in Biorender.

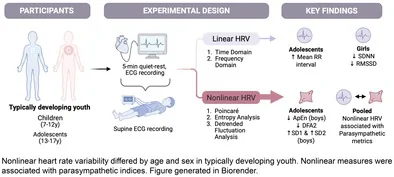

## 1 INTRODUCTION

Heart rate variability (HRV) is a measure of the fluctuations in the adjacent R‐R interbeat intervals in a given electrocardiogram (ECG) time series (Ernst, 2017; Shaffer et al., 2014; Shaffer & Ginsberg, 2017) and a well‐established biomarker of cardiac autonomic function. Importantly, reduction in HRV is a risk factor for cardiometabolic morbidity and mortality in older adults (Kaplan et al., 1991; Umetani et al., 1998; Yeragani et al., 1997) as well as various chronic diseases, e.g., heart failure (Camillo et al., 2008; Chattipakorn et al., 2007; Farah et al., 2014; Huikuri, 1995; Huikuri & Stein, 2012). Healthy aging is also associated with a significant and linear decrease in parasympathetic function and increase in sympathetic drive, starting as early as young adulthood (~20 years of age) (Jensen Urstad et al., 1997; Zhang, 2007). Age‐ and disease‐related reductions in HRV are largely driven by impairments in central autonomic network function, baroreflex sensitivity, endothelial function, and inflammation, to name a few (Thayer et al., 2010; Thayer et al., 2021). Sex may also influence HRV across the lifespan (Zaleski et al., 2023), such that females often demonstrate greater indices of parasympathetic modulation than males during young and middle adulthood. However, these differences appear to diminish with age, particularly after the female menopausal transition, potentially due to reductions in ovarian hormones estrogen and progesterone (Jensen Urstad et al., 1997; Koenig & Thayer, 2016; Schmalenberger et al., 2020; Zhang, 2007). These disease‐, age‐ and sex‐related differences highlight the importance of considering demographic factors when interpreting HRV.

However, a current limitation among the literature on HRV across the lifespan is the biological heterogeneity and methodological differences among studies. Similar challenges exist in pediatric populations, where interest in characterizing HRV development has grown as these periods encompass substantial physiological maturation. Interpretation from pediatric HRV findings are further complicated by methodological differences, such as recording length (e.g., long‐term, short‐term, or ultra‐short‐term) and the criteria used to categorize pediatric age groups—for example, the specific age at which childhood ends and adolescence begins (e.g., 10, 11, or 12 years). Nonetheless, cross‐sectional studies appear to demonstrate an increase in time and frequency domain measures of parasympathetic regulation of HRV and a decrease in cardiac sympathetic activity with age ranging from infancy to around 18 years of age (Bobkowski et al., 2017). Changes in HRV during childhood and adolescence appear to be driven by many of the same factors that influence HRV in other populations including the maturation of parasympathetic and cardiac function, (de Simone et al., 1992; Veijalainen et al., 2019) as well as physical activity, sleep, and psychoemotional stress (Billman et al., 2019; Herzig et al., 2017; Lins‐Filho et al., 2023; Speer et al., 2024). Although this age‐dependency of linear HRV during development has been for the most part well‐documented, (Galeev et al., 2002; Goto et al., 1997; Lenard et al., 2004; Massin & von Bernuth, 1997) the magnitude and age of peak HRV remains an area of discussion, with some studies reporting decreases in HRV (e.g., increased sympathetic activity and/or decreased parasympathetic regulation) (Finley & Nugent, 1995) starting at ~8 years of age, yet others report no change after early adolescence (~11 years old) (Fukuba et al., 2009; Silvetti et al., 2001). Whether linear HRV develops differently between young males and females also remains largely unexplored, despite the fact that puberty represents a critical period of neurohormonal maturation that may contribute to sexually dimorphic developmental trajectories of HRV (Billman et al., 2019; Bobkowski et al., 2017; Silvetti et al., 2001).

Furthermore, HRV is often analyzed in the framework of linear stochastic processes to provide information on the heart rate (HR) signal under stationary conditions (Lipsitz & Goldberger, 1992; Shaffer et al., 2014; Shaffer & Ginsberg, 2017). Linear HRV includes robust and well‐validated analyses based on statistical patterns (time domain) or power spectral analysis (frequency domain), but fails to account for the fact that the HR signal is influenced by multiple physiological systems under neural control and their interactions. In this way, the HR time series are inherently non‐stationary as HR continuously fluctuates in response to multiple physiological and environmental stimuli (Magagnin et al., 2011; Peng et al., 1995). Evidence from ambulatory Holter monitoring studies indicates that even brief periods of non‐stationarity can disproportionately influence HRV metrics, leading to an overestimation of sympathetic modulation (Magagnin et al., 2011). Consequently, complementary analytical approaches have gained increasing attention (Billman et al., 2019; Ernst, 2017; Lipsitz & Goldberger, 1992; Vaillancourt & Newell, 2002). Among these, methods adapted from chaos theory, including approximate entropy (ApEn) and detrended fluctuation analysis (DFA), are widely used to quantify the nonlinear complexity and fractal organization of HR dynamics (Beckers et al., 2006; Billman et al., 2019; Castiglioni et al., 2011; Perkiomaki et al., 2002; Willson et al., 2002). Aging is associated with reductions in the complexity and fractal dynamics of the nonlinear HR time series, (Pikkujamsa et al., 1999) and nonlinear HRV has shown to be a robust and independent measure in detecting abnormalities in HR (Voss et al., 2009) and predicting cardiac recovery and mortality risk (Stein et al., 2005) in disease (e.g., heart failure).

Still, little is known about the effects of childhood maturation on nonlinear HRV (Acharya et al., 2004; Cysarz et al., 2011; Kakavand et al., 2025; Kane et al., 2020; Pikkujamsa et al., 1999; Vanderlei et al., 2018). Cysarz et al. (2011) observed a peak in ApEn in 7–9‐year‐old children, decreasing thereafter and hitting a nadir in kids ≥14 years of age, suggesting that complexity is reduced in adolescents. In contrast, DFA was higher in the same group of adolescents, (Cysarz et al., 2011) indicating improved fractal dynamics in the HR time series compared with children. Pikkujamsa et al. (1999) documented a steady cross‐sectional decrease in ApEn from infancy up to ~80 years old. However, they reported a decrease in short‐term DFA in children and adolescents that began to increase again at mid‐life (~40 years of age) (Pikkujamsa et al., 1999).

Although limited previous studies suggest that nonlinear properties of HRV change throughout childhood and adolescence, the direction and magnitude of these changes vary across entropy‐ and fractal‐based metrics (Kakavand et al., 2025). Accordingly, the present study sought to cross‐sectionally characterize both short‐term linear and nonlinear HRV in a cohort of healthy, typically developing children (7–12 years old) and adolescents (13–17 years old). Puberty represents a critical period of cardiovascular and autonomic maturation, characterized by dynamic changes in circulating sex hormones that differ between males and females and may influence the developmental trajectory of cardiac autonomic regulation (Cysarz et al., 2011; Jarrin et al., 2015; Patton & Viner, 2007). However, whether linear and nonlinear indices of HRV develop differently in typically developing males and female children and adolescents remains incompletely understood (Cysarz et al., 2011; Kakavand et al., 2025). Therefore, we also performed an exploratory secondary analysis examining potential sex differences in HRV during development. Based on previous studies demonstrating age‐related increases in vagally mediated HRV indices during childhood and adolescence, (Billman et al., 2019; Bobkowski et al., 2017; Kakavand et al., 2025; Silvetti et al., 2001) we hypothesized that both linear and nonlinear HRV would be greater in adolescent than in children. Furthermore, because males undergo pronounced pubertal increases in cardiac size, stroke volume, and cardiorespiratory fitness, physiological adaptations that have been associated with higher HRV, (de Simone et al., 1992; de Simone et al., 1997; de Simone et al., 2001) we hypothesized that HRV would be greater in males than in females during development.

## 2 METHODS

### 2.1 Participants and study design

Data used in this manuscript are from two separate protocols within the same research laboratory approved by the Institutional Review Board at the University of Delaware (IRB Study No. 1097721, 1641284). Each study was conducted in accordance with the ethical standards of the Declaration of Helsinki and written informed consent was obtained from parents or legal guardians and written assent was obtained from the children and adolescents. Participants were recruited from the surrounding Newark, DE region. Participants were divided into groups by age as follows: child (ages 7–12 years old) and adolescent (ages 13–17 years old). Participants were typically developing and non‐obese, defined as having a body mass index (BMI) between the 5th and 95th percentiles for age, sex, and height. All participants were free of any history of chronic disease or conditions (e.g., autoimmune diseases, congenital diseases), free of sleep disorders or conditions known to affect sleep (e.g., depression), not taking medications or supplements that may alter blood vessel function or sleep, and did not have a recent history of coronavirus disease 2019 (SARS‐Cov‐2) infection (<60 days) or vaccination (<14 days). Following screening and consenting, participants returned to the laboratory on a separate day following a period of fasting (>6 h) and after having refrained from caffeine consumption and strenuous exercise and/or vigorous physical activity for 24 h.

### 2.2 Cardiac autonomic function

HRV was assessed as a surrogate of autonomic function of the heart following >10 min of quiet supine rest in a dimly lit room (Task Force of the European Society of Cardiology and the North American Society of Pacing and Electrophysiology, 1996). A three‐lead ECG was used to record beat‐to‐beat heart rate for 5 continuous minutes while participants breathed spontaneously. Consequently, respiratory frequency was not quantitatively recorded during the HRV assessment, and an influence of respiratory sinus arrhythmia cannot be ruled out. However, participants were continuously monitored during recording period and instructed at the start of the test to maintain a consistent breathing rate. Using a Lead II spot electrode configuration on the torso, surface electrodes were placed directly on the skin at three sites: (Shaffer & Ginsberg, 2017) upper right chest, (Shaffer et al., 2014) upper left chest, and (Ernst, 2017) lateral surface of the lower left abdomen. Electrodes were connected to a Biopac MP160 system (Biopac Systems, CA, USA) and the ECG signal sampled at a rate of 1000 Hz with an analog amplifier interfaced to the AcqKnowledge Version 5 software (Biopac Systems, CA, USA). Ectopics and artifacts were manually removed from the raw ECG file offline using Kubios HRV Scientific (Kubios Oy, Kuopio, Eastern Finland) (Alcantara et al., 2020), and the HRV data were excluded if >5 beats were corrected. The majority of assessments (54/64; 85%) were performed during the morning hours, although a small proportion of participants (10/64; 15%) were tested after 12:00 pm because of scheduling constraints.

Linear time domain metrics of HRV were calculated as the standard deviation of the normal sinus interbeat intervals (SDNN) and root mean square of the successive differences between the normal interbeat intervals (RMSSD). RMSSD and SDNN were retained in their original absolute units (ms) to preserve direct physiological interpretability and facilitate comparison with conventional values reported in the pediatric HRV literature. Using power spectral density (PSD) analysis, data in the time domain was transformed into the frequency domain using Fast Fourier Transformation (FFT), and frequency domain metrics of HRV were derived. Specifically, this allowed for calculation of very low frequency (VLF: 0.0033–0.4 Hz), low frequency (LF: 0.04–0.15 Hz) and high frequency (HF: 0.15–0.5 Hz) oscillations in absolute (ms^2^) and relative (percent [%] and normalized units [n.u.]) units, as well as total power. The LF and HF ratio was then calculated as an estimate of the autonomic balance between the oscillations of the sympathetic and parasympathetic branches, e.g., sympathovagal balance.

Complexity and fractal correlations were conducted to assess nonlinear metrics of HRV. Complexity was quantified using ApEn, a measure of system regularity, randomness, and predictability, which has been developed to work with a shorter and noisier time series characteristic of clinical data. ApEn was calculated according to the method proposed by Pincus, using an embedding dimension, *m*, set to 2, a tolerance radius, *r*, set to 0.2 × SDNN, and a data length, *N*, corresponding to the total number of normal sinus interbeat (NN) intervals contained within the analyzed 5‐min segment (Pincus, 1991). Conceptually, ApEn measures the likelihood that runs of R‐R time series of a given length (e.g., epochs) that are close will remain close with each succeeding incremental comparison. Thus, if the R‐R time series epochs are more frequent and similar (i.e., regular and non‐complex), ApEn is low, and if the R‐R time series is irregular and divergent (i.e., random and complex), ApEn is high. Sample Entropy (Sampen), a variation of ApEn, was also calculated as a complementary measure of complexity, (Francesco et al., 2012; Richman & Moorman, 2000) with parameters for the embedding dimension (*m*), tolerance radius (*r*), and data length (*N*) set to the same values as those used for ApEn. However, ApEn includes self‐matches in its calculations, whereas SampEn excludes self‐matches, making SampEn generally less biased and less dependent on data length (Pincus, 1991; Richman & Moorman, 2000). Consequently, SampEn aims to address potential limitations of ApEn, namely, Sampen is not biased by the record length of the time series and displays greater relative consistency (e.g., reliability), and thus the two measures do not always yield identical results, particularly when differences between groups are modest (Francesco et al., 2012; Pincus, 1991; Richman & Moorman, 2000).

DFA quantifies the long‐range temporal correlations and fractal properties of the R‐R interval time series. DFA was developed to quantify correlations embedded in nonstationary data, providing information on the scale‐invariant characteristics of the nonlinear time series. Described in detail by Peng et al., the root mean square of the integrated and detrended R‐R interval time series are calculated within a chosen window size, and then the log of the fluctuation plotted against the log of the window size (Peng et al., 1995). Log transformation reveals two separate linear relationships (i.e., slopes), namely, the scaling exponents *α*_1_ (short‐term scaling exponent; window size of 4–12 beats) and *α*_2_ (intermediate‐term scaling exponent; window size of 13–64 beats) (Peng et al., 1995). Although a 4–16‐beat range has also been used, the 4–12‐beat range was selected to maintain consistency with the analysis settings in the Kubios software and literature informing the present study. The intermediate‐term scaling exponent (DFA *α*2) was calculated over window sizes greater than 12 beats. Conceptually, the *α* exponent can be thought of as a measure of the “*roughness*” of the time series, where the larger the *α* exponent, the smoother the time series. An *α* value close to 0.5 reflects white noise (i.e., random, uncorrelated data), whereas values close to 1.5 reflect Brownian noise (i.e., highly correlated, rigid patterns). An *α* close to 1 reflects pink noise (i.e., fractal, self‐organized variability) and a compromise between the unpredictability of white noise and the rigid patterns of Brownian noise. Accordingly, an *α* close to 1 is associated with a healthier fractal HR dynamic.

Lastly, Poincaré analysis was conducted to characterize the HR dynamics using a nonlinear, two‐dimensional visual phase‐space representation in which each RR interval is plotted against the subsequent RR interval, allowing quantification of short‐ and long‐term variability through SD1 and SD2, respectively. First, sequential R‐R intervals were plotted against the next interval, creating a scattergram. Thus, each point in the Poincaré plot is described by two neighboring R‐R intervals, namely, the current R‐R interval (RR_*n*_) and the proceeding R‐R interval (RR_*n* + 1_). A line of identity was then fit, which is a theoretical linear regression slope where successive points of equal values fall along the 45° diagonal (i.e., RR_*n*_ = RR_*n* + 1_). An ellipse is fit on the scattergram overlaying the line of identity, which is used to calculate the dispersion of points perpendicular to the line of identity (short‐term variability; SD1) and along the line of identity (long‐term variability; SD2). Recent studies have demonstrated that SD1 is strongly associated with short‐term vagally mediated HRV and is mathematically equivalent to RMSSD up to a constant scaling factor and thus may better reflect linear HRV (Ciccone et al., 2017). SD2 reflects longer‐term R‐R interval variability and is influenced by multiple physiological mechanisms, including sympathetic autonomic regulation (Francesco et al., 2012). Although derived from a nonlinear geometric representation of R‐R interval dynamics, SD1 and SD2 are interpreted as nonlinear geometric of linear HRV (Shaffer et al., 2014).

### 2.3 Additional covariates

Pubertal status, BMI, and blood pressure (BP; i.e., systolic [SBP] and diastolic BP [DBP]) were collected on the same day as HRV assessment. BMI was calculated using standardized techniques and expressed as the ratio of weight (in kilograms) and height (in meters) squared. BMI was then expressed as a percentile and *z*‐score. The Tanner Pubertal Development questionnaire was used to characterize gonadarche and adrenarche pubertal development. Lastly, supine BP was assessed using a validated oscillometric Sphygmocor pulse wave analysis (PWA) system (SphygmoCor XCEL, AtCor Medical, Australia). BP percentiles were then calculated based on age and height percentiles. These covariates were selected on a theoretical basis, as typical development is characterized by gradual and consistent changes in cardiometabolic physiology, owing largely to the maturation of the neurohumoral system, as well as behavioral changes, and each of these variables is known to influence heart rate dynamics.

### 2.4 Statistical analysis

Statistical comparisons were performed using open‐source software (RStudio version 5.1.6, RStudio: Integrated Development for R, PBC, Boston, MA, USA). Tests of normality were conducted with the Shapiro–Wilk test and homogeneity of variance with the Levene test. However, to account for a modest total (*N* = 64) and sex‐by‐age subgroup sample size (*n* = 11–23), and uneven sample distribution between groups, non‐parametric analyses were performed where appropriate as they do not require the condition of normality. Two‐factor (age × sex) analysis of variance (ANOVA) was used to determine main effects of age (child vs. adolescent) and sex (male vs. female), and any potential age by sex interactions. When an interaction or main effect was found, post‐hoc two‐tailed Mann–Whitney *U* tests were performed on the individual observations using ranked data, with a Bonferroni‐Holm correction for multiple comparisons. Omega squared (*ω* ^2^) values were computed from the ANOVA, with magnitudes set at *ω* ^2^ = 0.01, *ω* ^2^ = 0.06, and *ω* ^2^ = 0.14 for small, medium, and large effect sizes, respectively (Cohen, 1988). We computed nonparametric common language effect sizes ($\hat{\theta }$) sizes for all between age or sex mean comparisons, with magnitudes set at θ = 56% for small, θ = 64% for medium, and θ = 71% for large effect sizes (McGraw & Wonf, 1992). Spearman Rank correlation was used for all correlation analysis, expressed as rho = *ρ*. Alpha was set, a priori, at 0.05 for all comparisons. For consistency with the pediatric HRV literature and to facilitate comparison with previous studies, descriptive data are presented as mean ± SD in the text and tables, whereas boxplots display the median, interquartile range, and mean (as filled squares).

## 3 RESULTS

### 3.1 Participant characteristics

A total of 64 apparently healthy typically developing children and adolescents were included in this data set. Group sample sizes and participant characteristics are presented in Table 1. As expected, adolescents were older, taller (child vs. adolescent, 142 ± 13 cm vs. 167 ± 11 cm, *p* < 0.001, *ω* ^2^ = 0.50), heavier (child vs. adolescent, 38 ± 12 kg vs. 60 ± 11 kg, *p* < 0.001, *ω* ^2^ = 0.50), had a higher non standardized BMI (child vs. adolescent, 18 ± 3 kg/m^2^ vs. 21 ± 3 kg/m^2^, *p* < 0.001, *ω* ^2^ = 0.27), and had a higher SBP (child vs. adolescent, 107 ± 9 mmHg vs. 114 ± 8 mmHg, *p* = 0.001, *ω* ^2^ = 0.15) (Table 1). However, when BMI, SBP, and DBP were expressed as percentiles, no interaction or main effects were observed.

**TABLE 1:** Participant characteristics.

	Child overall *N* = 35	Male child *n* = 23	Female child *n* = 12	Adolescent overall *N* = 29	Male adolescent *n* = 18	Female adolescent *n* = 11
Age (years)	10 ± 2	10 ± 2	9 ± 2	14 ± 2^a^	15 ± 2	14 ± 1
Height (cm)	142 + 13	143 ± 14	141 ± 12	167 ± 11^a^	171 ± 11	159 ± 7
Weight (kg)	38 ± 12	39 ± 13	35 ± 9	60 ± 11^a^	62 ± 11	59 ± 11
BMI (kg/m^2^)	18 ± 3	18 ± 3	17 ± 3	21 ± 3^a^	21 ± 2	23 ± 5
BMI (%)	55 ± 31	56 ± 31	52 ± 32	66 ± 25	61 ± 25	75 ± 24
SBP (mmHg)	107 ± 9	106 ± 9	107 ± 10	114 ± 8^a^	115 ± 9	114 ± 5
SBP (%)	60 ± 26	59 ± 27	61 ± 25	57 ± 23	51 ± 24	67 ± 16
DBP (mmHg)	67 ± 7	66 ± 6	67 ± 9	68 ± 7	69 ± 7	67 ± 6
DBP (%)	64 ± 19	64 ± 19	65 ± 22	58 ± 21	60 ± 22	56 ± 20
Tanner stage	1.72 ± 0.84	1.47 ± 0.52	2.10 ± 1.10	4.14 ± 0.83^a^	4.00 ± 0.96	4.38 ± 0.52

*Note*: BMI expressed as percentile.

Abbreviations: BMI, body mass index; DBP, diastolic blood pressure; SBP, systolic blood pressure.

^a^ Significant difference between children and adolescents, pooled.

### 3.2 Linear HRV

Time and frequency domain linear HRV metrics are presented in Table 2. Two‐way ANOVA did not reveal an interaction effect for the RR time‐interval (*p* = 0.620, *ω* ^2^ = 0.00). However, a main effect of age was found (*p* = 0.005, *ω* ^2^ = 0.10) such that adolescents had a higher RR interval than children (child vs. adolescent, 813 ± 114 ms vs. 896 ± 125 ms, *p* = 0.007, $\hat{\theta }$ = 70). Although a main effect of sex was not detected (*p* = 0.078, *ω* ^2^ = 0.03), it was trending towards a lower RR time interval in females when data were pooled across children and adolescents.

**TABLE 2:** Time and frequency domain linear HRV.

	Male child *n* = 23	Male adolescent *n* = 18	Female child *n* = 12	Female adolescent *n* = 11
RR Interval (ms)	826 ± 123	923 ± 133^b^	786 ± 90	852 ± 102^b^
SDNN (ms)	70 ± 34	68 ± 24	68 ± 29^a^	40 ± 14^a^
RMSSD (ms)	85 ± 42	80 ± 34	72 ± 31^a^	48 ± 24^a^
VLF Power (ms^2^)	191 ± 277	86 ± 85^b^	220 ± 395	45 ± 33^b^
VLF Power (%)	3.79 ± 3.87	2.15 ± 2.04	3.74 ± 4.30	3.61 ± 2.34
LF Power (ms^2^)	2035 ± 2338	1616 ± 1831	2018 ± 2346	483 ± 297
LF Power (%)	35 ± 14	40 ± 22	38 ± 16	34 ± 15
LF Power (n.u.)	37 ± 13	41 ± 22	40 ± 15	36 ± 15
HF Power (ms^2^)	2895 ± 2338	2681 ± 1831	2772 ± 4114	941 ± 792
HF Power (%)	62 ± 13	58 ± 22	58 ± 16	62 ± 15
HF Power (n.u.)	65 ± 13	59 ± 22	60 ± 15	65 ± 15
LF/HF	0.62 ± 0.39	1.07 ± 1.25	0.80 ± 0.64	0.67 ± 0.62

*Note*: Significance denotes two‐tailed Mann–Whitney *U* test with a Bonferroni post hoc comparison.

Abbreviations: HF, high frequency; LF/HF, ratio of the LF to HF; LF, low frequency; RMSSD, root mean square of successive differences between normal heartbeats; SDNN, standard deviation of the normal sinus beats (SDNN); VLF, very low frequency.

^a^ Significant difference between males and females, pooled.

^b^ Significant difference between child and adolescent, pooled.

For time domain indices of HRV (i.e., SDNN and RMSSD), two‐way ANOVA did not reveal any interaction effect (*p* > 0.05) or a main effect of age (*p* > 0.05). However, a main effect of sex for SDNN (*p* = 0.048, *ω* ^2^ = 0.05) and RMSSD (*p* = 0.021, *ω* ^2^ = 0.07) was observed, such that females had lower SDNN (female vs. male, 55 ± 27 ms vs. 70 ± 30 ms, *p* = 0.040, $\hat{\theta }$ = 66) and RMSSD (female vs. male, 61 ± 30 ms vs. 83 ± 38 ms, *p* = 0.019, $\hat{\theta }$ = 68) as compared to males when data were pooled across children and adolescents. For frequency domain indices of HRV (i.e., VLF, LF, HF), two‐way ANOVA did not reveal any interaction effect, or a main effect of age or sex (*p* > 0.05).

### 3.3 Nonlinear HRV: Approximate and sample entropy

Two‐way ANOVA revealed a significant interaction effect of age by sex (*p* = 0.010, *ω* ^2^ = 0.09) for ApEn (Figure 1a). Post‐hoc analysis revealed significantly lower ApEn in adolescent males as compared to children (child vs. adolescent, 1.18 ± 0.07 a.u. vs. 1.07 ± 0.08 a.u., *p* < 0.001, $\hat{\theta }$ = 86). Two‐way ANOVA did not reveal an interaction effect (*p* = 0.212, *ω* ^2^ = 0.00) or main effects for age (*p* = 0.516, *ω* ^2^ = 0.00) and sex (*p* = 0.108, *ω* ^2^ = 0.03) for Sampen (Figure 2b).

**FIGURE 1:**
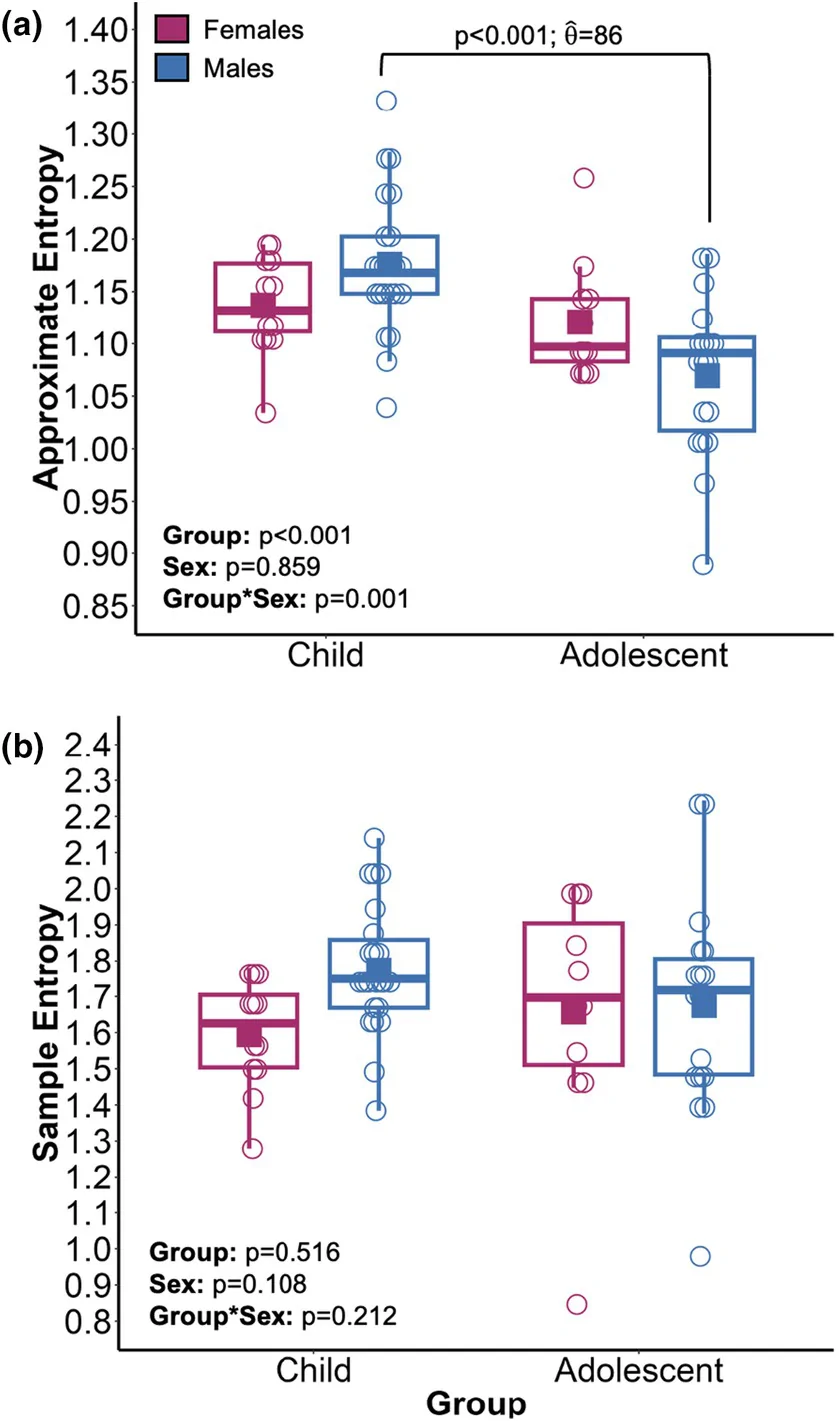
Complexity measures of nonlinear HRV. (a) Approximate entropy. (b) Sample entropy. Solid squares in box plots denote the sample mean. *p*‐values in brackets denote two‐tailed Mann–Whitney *U* test with a Bonferroni post hoc comparison for child males versus adolescent males. CLES ($\hat{\theta }$); common language effect size. Child Females: *N* = 12, Adolescent Females: *N* = 11, Child Males: *N* = 23, Adolescent Males: *N* = 18.

**FIGURE 2:**
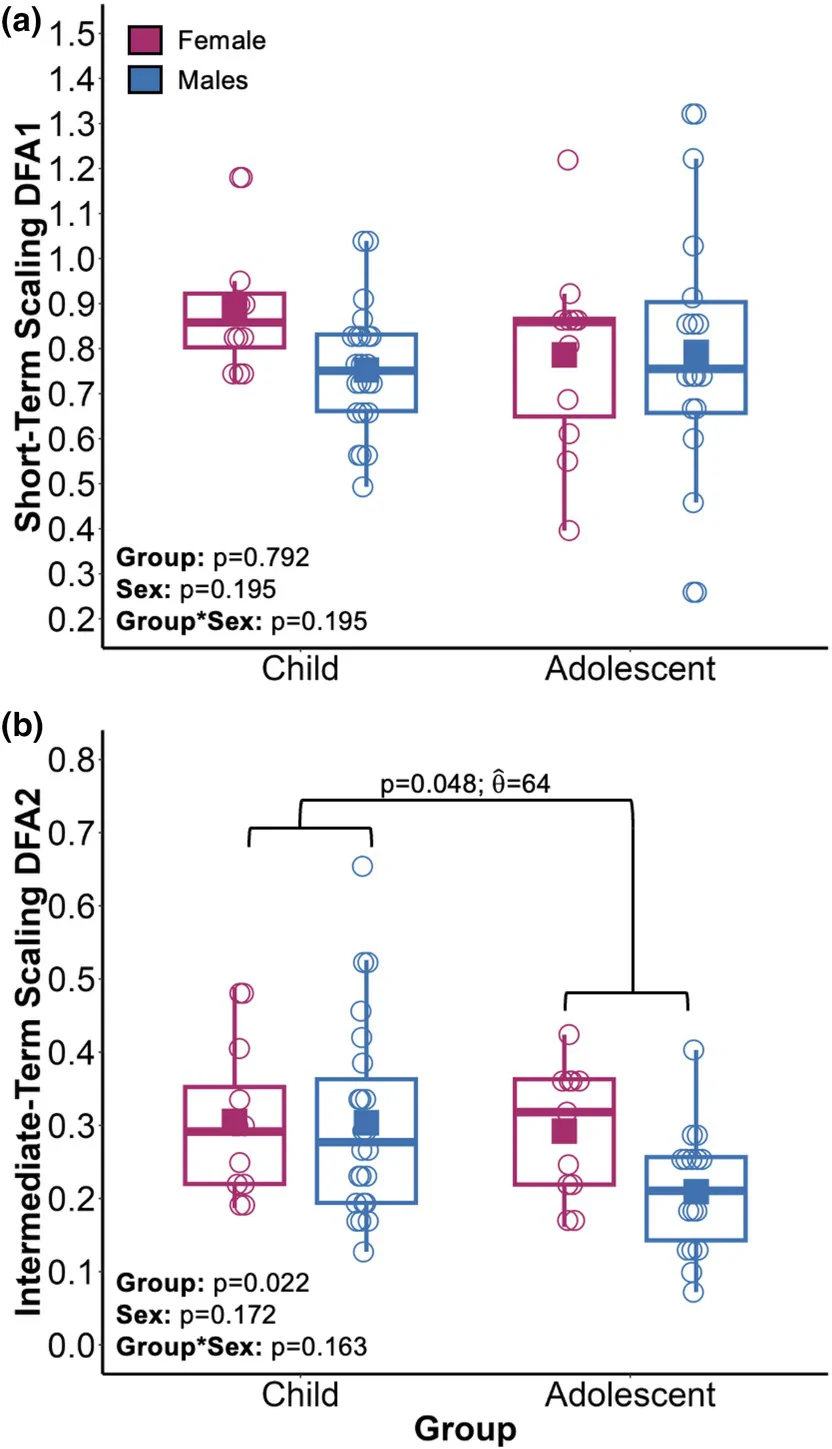
Detrended fluctuation measures of nonlinear HRV. (a) Short‐term scaling exponent *α*_1_. (b) Intermediate‐term scaling exponent *α*_2_. Solid squares in box plots denote the sample mean. *p*‐values in brackets denote two‐tailed Mann–Whitney *U* test with a Bonferroni post hoc comparison for child versus adolescent, pooled across sex. CLES ($\hat{\theta }$); common language effect size. Child Females: *N* = 12, Adolescent Females: *N* = 11, Child Males: *N* = 23, Adolescent Males: *N* = 18.

### 3.4 Nonlinear HRV: Detrended fluctuation analysis

Two‐way ANOVA did not reveal an interaction effect (*p* = 0.195, *ω* ^2^ = 0.01) for the short‐term scaling exponent, DF*α*_1_ (Figure 2a). Likewise, no main effect of age (*p* = 0.792, *ω* ^2^ = 0.00) or sex (*p* = 0.195, *ω* ^2^ = 0.01) was observed. Two‐way ANOVA did not reveal an interaction effect (*p* = 0.163, *ω* ^2^ = 0.02) for the intermediate‐term scaling exponent, DF*α*_2_ (Figure 2b). A main effect of age was confirmed (*p* = 0.022, *ω* ^2^ = 0.07), although there was no main effect of sex (*p* = 0.172, *ω* ^2^ = 0.01). Post‐hoc analysis revealed a lower intermediate‐term DFA*α*_2_ in adolescents as compared to children (child vs. adolescent, 0.30 ± 0.12 a.u. vs. 0.24 ± 0.09 a.u., *p* = 0.048, $\hat{\theta }$ = 64).

### 3.5 HRV: Poincáre analysis

Two‐way ANOVA did not reveal an interaction effect (*p* = 0.321, *ω* ^2^ = 0.00) for short‐term variability, SD1 (Figure 3a). There was no main effect of age (*p* = 0.191, *ω* ^2^ = 0.01), although a significant main effect of sex was observed (*p* = 0.021, *ω* ^2^ = 0.07). Post‐hoc analysis revealed a significantly higher SD1 for males compared to females when data were pooled across children and adolescents (female vs. male, 43 ± 21 ms vs. 59 ± 27 ms, *p* = 0.019, $\hat{\theta }$ = 68).

**FIGURE 3:**
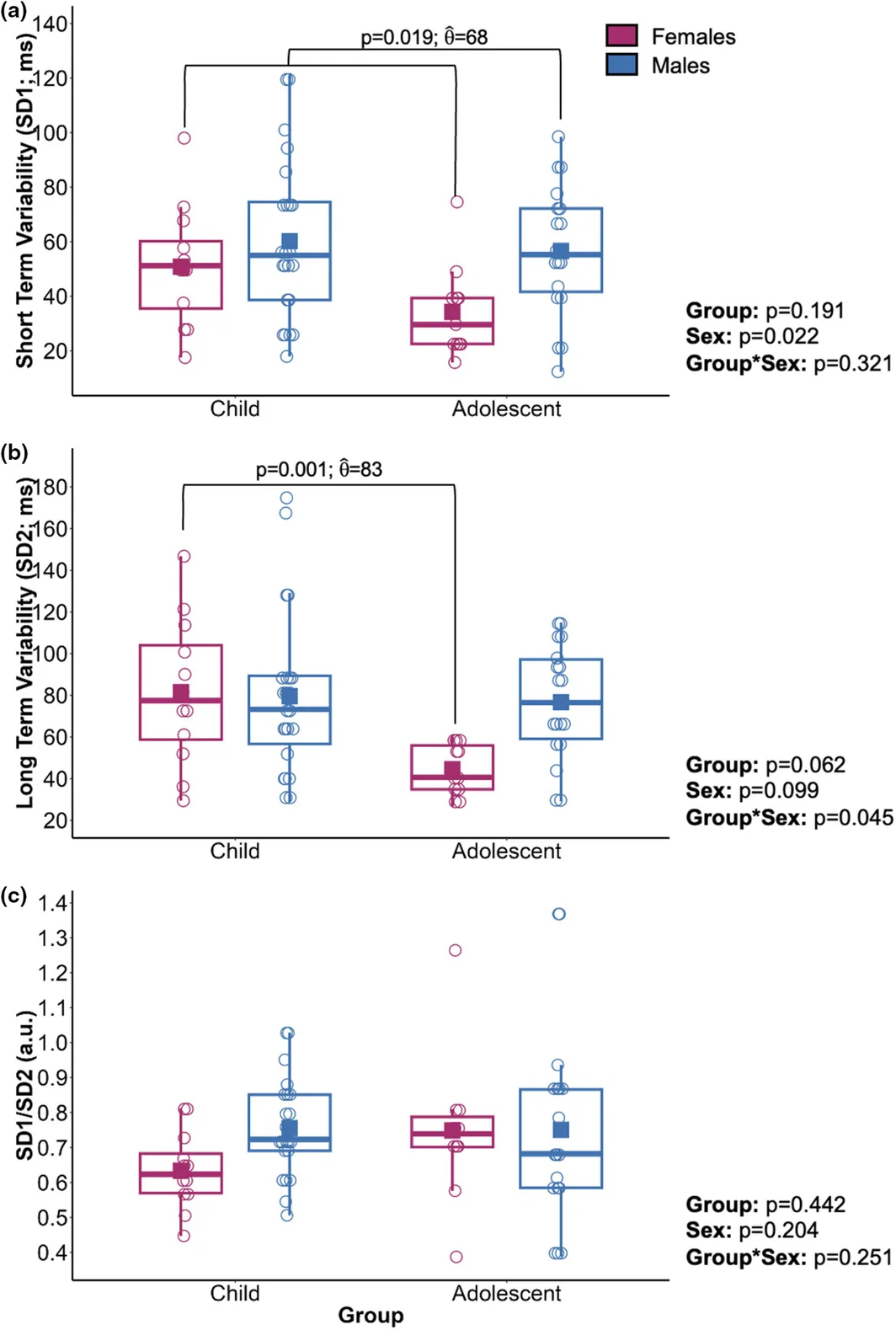
Poincare plot measures of nonlinear HRV. (a) Short‐term variability (SD1). (b) Long‐term variability (SD2). (c) Ratio of SD1 to SD2. Solid squares in box plots denote the sample mean. *p*‐values in brackets denote two‐tailed Mann–Whitney *U* test with a Bonferroni post hoc comparison for males versus females, pooled across age. CLES ($\hat{\theta }$); common language effect size. Child Females: *N* = 12, Adolescent Females: *N* = 11, Child Males: *N* = 23, Adolescent Males: *N* = 18.

Two‐way ANOVA revealed an interaction effect (*p* = 0.046, *ω* ^2^ = 0.05) for long‐term variability, SD2 (Figure 3b). Post‐hoc analysis revealed significantly lower SD2 in adolescent females as compared to children (child vs. adolescent, 81 ± 35 ms vs. 45 ± 12 ms, *p* = 0.011, $\hat{\theta }$ = 83). There was no main effect of age (*p* = 0.062, *ω* ^2^ = 0.04) or main effect of sex (*p* = 0.099, *ω* ^2^ = 0.03), although it was trending.

Lastly, two‐way ANOVA did not reveal an interaction effect (*p* = 0.251, *ω* ^2^ = 0.00), main effect of age (*p* = 0.442, *ω* ^2^ = 0.00), or main effect of sex (*p* = 0.204, *ω* ^2^ = 0.01) for the ratio of SD1 and SD2 (Figure 3c).

### 3.6 Correlations between linear and nonlinear heart rate variability

Correlations between short‐term nonlinear and linear HRV metrics are presented in Table 3. ApEn was negatively correlated with the mean RR time‐interval (*ρ* = −0.55) and the LF/HF ratio (*ρ* = −0.34). Sampen was positively correlated with RMSSD (*ρ* = 0.21), relative HF power (*ρ* = 0.29), and negatively correlated with the LF/HF ratio (*ρ* = −0.39). DF*α*_1_ was negatively correlated with RMSSD (*ρ* = −0.36), relative HF power (*r* = −0.83) but positively correlated with the LF/HF ratio (*ρ* = 0.74). DF*α*_2_ was negatively correlated with the mean RR time‐interval (*ρ* = −0.49), SDNN (*ρ* = −0.38), RMSSD (*r* = −0.47), absolute (*ρ* = −0.47) and relative HF power (*ρ* = −0.31), and total frequency power (*ρ* = −0.34). SD1 was positively correlated with the mean RR time‐interval (*ρ* = 0.58), SDNN (*r* = 0.94), RMSSD (*r* = 0.98), absolute HF power (*r* = 0.92) and total frequency power (*r* = 0.85). Likewise, SD2 was also positively correlated with the mean RR time‐interval (*ρ* = 0.44), SDNN (*r* = 0.98), RMSSD (*r* = 0.85), absolute HF power (*r* = 0.87) and total power (*r* = 0.94). Lastly, the ratio of SD1/SD2 was positively correlated with the mean RR time‐interval (*ρ* = 0.39), RMSSD (*ρ* = 0.46), absolute (*ρ* = 0.24) and relative (*ρ* = 0.69) HF power, and negatively correlated with the LF/HF ratio (*ρ* = −0.59).

**TABLE 3:** Correlation between linear and nonlinear HRV.

	RR interval	SDNN	RMSSD	HF power (ms^2^)	HF power (%)	Total power (ms^2^)	LF/HF
ApEn	−0.55	N/A	N/A	N/A	N/A	N/A	−0.34
SampEn	N/A	N/A	0.21	N/A	0.29	N/A	−0.39
DFA1	N/A	N/A	−0.36	N/A	−0.83	N/A	0.74
DFA2	−0.49	−0.38	−0.47	−0.47	−0.31	−0.34	N/A
SD1	0.58	0.94	0.98	0.92	N/A	0.85	N/A
SD2	0.44	0.98	0.85	0.87	N/A	0.94	N/A
SD1/SD2	0.39	N/A	0.46	0.24	0.69	N/A	−0.59

*Note*: All rho values denote correlations that were significant. N/A denotes no significant correlations.

Abbreviations: ApEn, approximate entropy; DFA1, detrended fluctuation analysis short term scaling exponent; DFA2, detrended fluctuation analysis long term scaling exponent; HF, high frequency; LF/HF, ratio of the LF to HF; LF, low frequency; RMSSD, root mean square of successive differences between normal heartbeats; SampEn, sample entropy; SD1, standard deviation (SD) of the instantaneous beat‐to‐beat interval variability; SD2, SD of the continuous long‐term beat‐to‐beat interval variability; SDNN, standard deviation of the normal sinus beats (SDNN); VLF, very low frequency.

## 4 DISCUSSION

The present study characterized short‐term linear and nonlinear HRV in a modest sample cohort of healthy, typically developing children and adolescents. A higher mean RR time‐interval (i.e., reduction in HR) was observed in the adolescent group. Unexpectedly, this did not correspond to a difference in other linear HRV measures between the adolescent group and child group. Males demonstrated a higher linear HRV than females, indicating a potential sex difference in cardiac autonomic function in youth. The adolescent group demonstrated increased regularity in the nonlinear HRV dynamics compared with children, a change that was especially evident in males. DFA revealed a reduced fractal dynamic in the adolescent group compared with children. Sex differences were also observed from Poincáre analysis, with males having higher short‐ and long‐term variability, a difference which appeared to be amplified in adolescents. Most of our nonlinear metrics were correlated with linear indices of parasympathetic tone. Collectively, our data demonstrate unique differences in short‐term linear and nonlinear HRV metrics between children versus adolescents, and males versus females, with some evidence of age by sex interactions.

### 4.1 Linear HRV

Linear HRV decreases steadily with age and is marked by a sharp decline in vagal tone, such that there is a shift towards greater cardiac sympathetic activity in old age (Jensen Urstad et al., 1997; Zhang, 2007). Contrary, a large body of literature reports positive associations between linear HRV and age during childhood development; (Bobkowski et al., 2017; Galeev et al., 2002; Goto et al., 1997; Lenard et al., 2004; Massin & von Bernuth, 1997) but this relation appears to be non‐uniform and non‐monotonic (i.e., increasing, plateauing, and decreasing at different stages of development) (Galeev et al., 2002). For example, parasympathetic activity appears to increase during development, but peak around early‐to‐middle adolescence (~15 years of age) (Bobkowski et al., 2017; Galeev et al., 2002). Using ambulatory 24‐h recording, a separate study found that linear HRV peaks at ~8–10 years of age, (Silvetti et al., 2001) while others reported a plateau in linear HRV as early as 6 years of age (Goto et al., 1997) and even a slight decline starting in late adolescence (Finley & Nugent, 1995). In the present cross‐sectional study, a higher RR interval (i.e., decreased HR) was observed among the adolescent group, but this did not correspond to differences in linear HRV (e.g., RMSSD, SDNN, and HF power). In contrast to the short, 5‐min recording paradigm used in the present study, most studies use 24‐h ambulatory ECG Holter monitoring, which captures other factors that influence HRV (e.g., physical activity, circadian rhythms) and may explain the discrepancies in our results. Additionally, the use of a narrow age‐range (7–17 years old) and the potential for parasympathetic nervous system (PNS) saturation, whereby further increases in vagal tone may not be reflected in time‐ or frequency‐domain HRV indices, (Goldberger et al., 2001) may present additional confounders. Interestingly, a recent study by Kakavand et al. (2025), which included participants aged 1–18 years, reported age‐related increases in RMSSD, SDNN, and frequency‐domain measures such as HF and LF power using 24‐h Holter monitoring, emphasizing the need for more short‐term HRV studies in children and adolescents. Additionally, complementary nonlinear HRV metrics that capture more complex autonomic dynamics, e.g., measures of nonlinear complexity, may offer greater sensitivity to deciphering the underlying differences in cardiac autonomic activity during development. Collectively, the cross‐sectional lower HR observed in the adolescent group compared with children is likely attributed to the development of other cardiovascular regulatory systems, for example, an increase in cardiac size and stroke volume, and not strictly changes in neurocardiac function (de Simone et al., 1992; de Simone et al., 1997), which requires future research.

Sex differences were observed for linear measures of HRV, i.e., SDNN and RMSSD (Table 2), such that linear HRV was higher in males. Our findings add to previous literature that failed to document sex differences in cardiac autonomic function during development, and is in agreement with studies that show a higher SDNN, and thus overall variability, in males during childhood and early adolescence (Galeev et al., 2002; Silvetti et al., 2001). A shift in the relative contribution of sympathetic and parasympathetic tone has also been reported during late adolescence, such that females present with higher vagal tone and higher linear HRV. This improved sympathovagal balance persists into young adulthood, but regresses during the menopausal transition and post‐menopause (Jensen Urstad et al., 1997; Koenig & Thayer, 2016; Zhang, 2007). However, we did not observe a higher RMSSD or HF power (both markers of vagal tone) in our cohort of females, but rather, adolescent males demonstrated greater RMSSD. This may be partly explained by the younger age of our adolescent female participants (14 ± 2 years old), as sex differences may become more pronounced after 20 years of age in females. Indeed, Galeev et al. (2002) found no sex difference between 16‐year‐old male and female adolescents, (Galeev et al., 2002) while Silvetti et al. (2001) saw improvements in SDNN in 18–20‐year‐old females (Silvetti et al., 2001). Consistent with this, the LF to HF ratio, a crude marker of sympathovagal balance, and the SD1 to SD2 ratio, a nonlinear index of sympathovagal balance, was not different between males and females in the present study. Although their validity as markers of sympathovagal balance is an area of debate, (Shaffer et al., 2014; Shaffer & Ginsberg, 2017) this may indicate minimal to no cross‐sectional differences in the relative contribution of vagal tone based on sex in typically developing kids between the ages of 7–17 years old.

Lastly, Poincaré plot analysis revealed lower SD1 and SD2 values in females compared with males. These findings mirrored the lower RMSSD and SDNN values observed in females, which is not surprising given the close mathematical and physiological relationships among these indices. Specifically, SD1 is directly related to RMSSD (SD1 = RMSSD/√2) and reflects short‐term, predominantly vagally mediated HRV (Ciccone et al., 2017). Accordingly, SD1 was strongly correlated with RMSSD in our cohort of kids and adolescents (Table 3). Similarly, the strong correlation between SD2 and SDNN suggests that both metric capture related aspects of overall HRV. Collectively, these findings indicate that the Poincaré‐derived measures largely corroborate the linear HRV results while providing a complementary nonlinear geometric characterization of sex differences in cardiac autonomic regulation (Ciccone et al., 2017). These observations are consistent with previous studies reporting sex differences in linear HRV development (Galeev et al., 2002; Silvetti et al., 2001). Although Bobkowski reported no sex difference in SD1 and SD2, they also failed to detect sex differences in linear HRV findings (Bobkowski et al., 2017). Interestingly, adolescent females exhibited numerically higher SD1 and SD2 values than female children, suggesting a potential maturation of autonomic regulation across development, although these differences did not reach statistical significance. Taken together, these findings suggest that age‐ and sex‐related influences on cardiac autonomic regulation may extend to both linear and nonlinear measures of HRV, warranting further investigation in larger longitudinal studies.

### 4.2 Nonlinear HRV

Less is known about the nonlinear dynamics of HRV during development (Acharya et al., 2004; Cysarz et al., 2011; Kakavand et al., 2025; Massin & von Bernuth, 1997; Pikkujamsa et al., 1999). Using a cross‐sectional study design and 24‐h Holter monitoring, Cysarz et al. (2011) observed a peak in ApEn in 7–9‐year‐olds, with an added effect of sex with age such that males >13 years had a higher ApEn than females (Cysarz et al., 2011). Pikkujamsa et al. (1999) also reported a gradual and monotonic cross‐sectional decrease in ApEn using 24‐h Holter monitoring across the lifespan (infancy to 80 years of age), although the decrease was not significant until middle age (~40 years of age) (Pikkujamsa et al., 1999). More recently, a study of typically developing children ranging in age from 1 day to 18 years reported that both SampEn and ApEn decreased with declining HR, indicating greater signal regularity and lower complexity of HRV dynamics. In contrast, Shannon entropy increased as HR decreased, suggesting an opposing pattern of greater complexity at lower heart rates (Kakavand et al., 2025). Likewise, we cross‐sectionally observed a lower ApEn in the adolescent group compared with children in the present study, suggesting an increase in the regularity of the nonlinear HR time series. Post hoc analyses further revealed that the reduction in ApEn in the adolescent group was largely driven by a more uniform HR dynamics in males. It is important to note that we did not observe any differences in SampEn, similar to the Shannon entropy results of Kakavand et al. (2025). Although ApEn and SampEn both quantify signal regularity (i.e., inverse of complexity), they differ in their computational approach, particularly with respect to the treatment of self‐matches (Pincus, 1991; Richman & Moorman, 2000). Consequently, these metrics may demonstrate differing sensitivities to developmental changes in HRV. However, the observed discrepancy between ApEn and SampEn may also reflect artifact related to differential parameterization. Thus, although the ApEn comparison reached statistical significance, the direction of the effect was not reflected in SampEn, necessitating future investigation on the developmental trajectory of HRV complexity (Kakavand et al., 2025).

DFA revealed greater uncorrelated randomness (*α* close to 0.5) in the intermediate scaling exponent in the adolescent group. Interestingly, females appeared to maintain a short‐term scaling exponent that better resembled fractal self‐organized variability (*α* close to 1). This is in contrast to findings from 24‐h HRV studies where Cysarz et al. (2011) documented improvements in the fractal dynamics of the HR signal in adolescence (*α* close to 1), but in agreement with Kakavand et al. (2025), who documented decreases in fractal detrended fluctuation, and thus, diminishing self‐similarity, across development until 18 years of age. Pikkujamsa et al. (1999), however, documented no change in DFA between children (<15 years old) and young adults (15–39 years old). A U shape response was observed in the study by Pikkujamsa et al. (1999) study, such that intermediate‐term fractal correlations decreased during adolescence and increased again during early adulthood.

The adolescent group demonstrated lower ApEn, indicating greater regularity of the HR time series, and an intermediate‐term DFA scaling exponent closer to 0.5, indicating weaker temporal correlations. ApEn and DFA characterize distinct properties of HR dynamics: ApEn quantifies pattern regularity, whereas DFA evaluates the correlation structure across temporal scales. Therefore, greater regularity can coexist with weaker temporal correlations. The physiological significance of this combination during development remains uncertain and necessitates future research. Lipsitz and Goldberger (1992) originally proposed the theory of loss of complexity in physiological systems in aging and disease (Lipsitz & Goldberger, 1992; Pikkujamsa et al., 1999; Stein et al., 2005; Vaillancourt & Newell, 2002; Voss et al., 2009). However, the authors did not address childhood and development, raising questions as to its applicability in pediatric cohorts. Given that complexity reflects the interaction of regulatory systems (e.g., the crosstalk between hemodynamic and neural systems), the direction and pattern of change in complexity may depend on alterations in the intrinsic dynamics of these systems, which are influenced by environmental demands (Vaillancourt & Newell, 2002) that change during development (e.g., puberty). Thus, ‘*irregularity*’/‘*randomness*’ and ‘*complexity*’ may not be synonymous during development (Stein, Domitrovich, Hui, et al., 2005). For example, an erratic and irregular HR structure in childhood may not offer the same level of adaptability as a dynamically complex system (Beckers et al., 2001). Nevertheless, this remains speculative and future mechanistic insights into nonlinear HRV during development are therefore warranted.

Despite no cross‐sectional difference in linear HRV between kids and adolescents, we observed that DFA was moderately and negatively correlated to RMSSD and HF power, and positively to the LF/HF ratio, established markers of vagal tone and sympathovagal balance, respectively. A similar, albeit weaker, negative relation between ApEn and SDNN was also observed. These associations indicate that linear and nonlinear HRV measures may capture related aspects of heart‐rate dynamics during development (Masten, 2004; Patton & Viner, 2007). This is partly reflected in the lower HR in the adolescent group, which may indicate maturation of sinus‐mediated regulation of cardiac function (e.g., setting HR to a basal value of ~60–80 bpm). Interestingly, in healthy older adults, changes in RR interval length following vagal stimulation does not appear to mirror nonlinear changes in HRV (Cepeda et al., 2018). It is therefore conceivable that maturation of vagal systems may play a role in regulating both linear and nonlinear HR dynamics in kids and adolescents. Autonomic regulation undergoes substantial changes during puberty, and specifically, an increase in sympathetic activity and changes in vagal modulation with advancing pubertal stage could contribute to difference in HRV profiles irrespective of age. Mechanistic studies further support this notion, as an acute dose of the muscarinic antagonist atropine increases scaling exponents from uncorrelated randomness (*α* = 0.5) and fractal scaling (*α* = 1) towards self‐similarity (*α* = 1.5), (Castiglioni et al., 2011) and passive head‐up tilt (Tulppo et al., 2001) and high intensity exercise (Tulppo et al., 1996) (both models of sympathetic outflow) increase ApEn and DFA. Importantly, given the cross‐sectional design, these findings cannot establish whether maturation of autonomic regulation contributes to the observed nonlinear HRV patterns. Developmental changes in autonomic, cardiovascular, hormonal, or other regulatory processes represent possible mechanisms that require evaluation in longitudinal and mechanistic studies.

### 4.3 Experimental considerations

Important experimental considerations must be addressed to contextualize the present findings. First, the present study is cross‐sectional in design, meaning that associations observed between variables cannot be interpreted as causal relationships. Second, the modest overall sample size (*N* = 64) constrained the ability to achieve balanced representation across sex‐by‐age subgroups (e.g., *n* = 12 for female child and *n* = 12 for female adolescent groups), limiting statistical power. Although this may increase the risk of Type I errors (e.g., false positives), non‐parametric *t*‐tests and statistical models that account for uneven and skewed distributions were used, and effect sizes of moderate to large magnitude are reported. Third, respiratory rate was not controlled in the present study. Because respiration acutely influences HRV, particularly, indices of parasympathetic modulation such as RMSSD and high‐frequency power, variations in breathing frequency and amplitude may have altered beat‐to‐beat heart rate dynamics via respiratory sinus arrhythmia, representing a potential confound. However, participants were monitored throughout the 5‐min recording, with standardized instructions provided to ensure a consistent respiratory rate across the testing period. Fourth, the role of sex hormones needs to be further explored for more precise analysis of the role of sex during development. For example, future studies should incorporate detailed menstrual cycle history questionnaires. Fifth, HRV indices in pediatrics have been shown to be influenced by resting HR (Gasior et al., 2018). Although conventional HRV measures were used to maintain consistency with previous pediatric studies, future investigations incorporating heart rate‐corrected HRV metrics may help distinguish mathematical from physiological contributions to developmental differences in HRV (Kakavand et al., 2025). Sixth, although the majority of ECG recordings were obtained during the morning, approximately 15% of participants (10/64) were assessed after 12:00 pm because of scheduling constraints. Given the known influence of circadian rhythms on HRV, the inclusion of a small number of afternoon assessments may have contributed modestly to variability in the HRV measurements. Lastly, we did not use 24‐h Holter ECG monitoring, considered the *‘gold‐standard’* assessment of cardiac autonomic function. 24‐h Holter ECG may better capture autonomic nervous responses to environmental factors (e.g., physical activity and circadian rhythms) and may explain some of the differing results between the present study and the literature. However, HRV recordings of ≤5‐min durations under controlled conditions have been validated as an assessment of cardiac autonomic function that is not flawed by movement and respiratory‐linked biases (Task Force of the European Society of Cardiology and the North American Society of Pacing and Electrophysiology, 1996). They present external validity in pediatric populations and may complement 24‐h Holter monitoring in clinical pediatric patients. This is enticing, as the nonlinear structure of the HR time series is a promising subclinical biomarker of pathology in older adults that remains poorly understood in pediatric disorders and has yet to be fully characterized using short‐term paradigms.

### 4.4 Conclusion

To the best of our knowledge, this is the first study to characterize spontaneous breathing, short‐term (5 min) nonlinear HRV during childhood development. We revealed unique differences in short‐term nonlinear HRV between sex‐by‐age subgroups not captured by linear analysis. The adolescent group was characterized by an increase in the regularity of the nonlinear HR dynamics, with loss of the fractal dynamics of the HR time series, specifically, the intermediate scaling DFA *α*2, compared with the children group. Furthermore, sex differences were observed, such that males had a more irregular nonlinear HR dynamics, uncorrelated randomness, and higher short‐ and long‐term variability as assessed by Poincáre analysis compared with females. Although preliminary, associations between nonlinear indices and conventional linear HRV measures suggest that these metrics may reflect partially related features of HR dynamics; however, the underlying physiological mechanisms cannot be determined from the present cross‐sectional data. Short‐term nonlinear HRV is therefore gaining clinical interest as an enticing, complementary assessment of cardiac autonomic function in pediatrics, however, it remains exploratory. Future work is warranted into the physiological mechanisms driving short‐term nonlinear HRV during development, and how it is affected in childhood disorders.

## AUTHOR CONTRIBUTIONS

**Alexs A. Matias:** Data curation; formal analysis; investigation; project administration; visualization. **Michele N. D'Agata:** Data curation; investigation; project administration. **Krista M. Szymanski:** Data curation; investigation; project administration. **Evan C. Ciecko:** Data curation; investigation; project administration. **Melissa A. Witman:** Conceptualization; data curation; formal analysis; funding acquisition; investigation; visualization.

## FUNDING INFORMATION

This study was supported in part by National Institutes of Health grant P20GM113125 and R01HLI557644.

## CONFLICT OF INTEREST STATEMENT

The authors declare that the research was conducted in the absence of any commercial or financial relationships that could be construed as a potential conflict of interest.

## ETHICS STATEMENT

This study was reviewed and approved by the Human Subjects Institutional Review Board at the University of Delaware (IRB #1097721, IRB #1641284) and was conducted in accordance with the Declaration of Helsinki. Written informed consent was obtained from parents or legal guardians and written assent from the children and adolescents.

## Data Availability

The datasets generated and/or analyzed during the current study are available from the corresponding author on reasonable request. *Code Availability*: The code used to support the findings of this study is available from the corresponding author on reasonable request.
